# Establishment of a Fosmid Library for Pseudorabies Virus SC Strain and Application in Viral Neuronal Tracing

**DOI:** 10.3389/fmicb.2020.01168

**Published:** 2020-06-11

**Authors:** Hansong Qi, Hongxia Wu, Muhammad Abid, Hua-Ji Qiu, Yuan Sun

**Affiliations:** State Key Laboratory of Veterinary Biotechnology, Harbin Veterinary Research Institute, Chinese Academy of Agricultural Sciences, Harbin, China

**Keywords:** pseudorabies virus, fosmid library, UL36, neuron, transport

## Abstract

Pseudorabies virus (PRV) is a member of *Alphaherpesvirinae* subfamily, its neurotropism and latency infection attract the attention of many scientists. PRV tagged with a fluorescent reporter gene as a tracker has been used to analyze neuronal circuits, including anterograde and retrograde. In this study, we used fosmid library to construct a rapid and efficient platform to generate recombinant PRV. Firstly, the highly purified PRV ShuangCheng (SC) genomic DNA was sheared randomly into approximately 30–49-kb DNA fragments. After end-blunting and phosphorylation, the DNA fragments were cloned into the fosmid vector and transformed into *Escherichia coli*. A total of 200 fosmids that cover the complete genome of PRV SC was sequenced. Thirteen fosmid combinations in five groups were transfected into Vero cells, respectively, and each group can successfully rescue PRV strain SC. There was no significant difference between wild type and recombinant in both morphology and growth kinetics. In the next step, an enhanced green fluorescent protein (EGFP) was fused into the amino-terminal of UL36 protein by Red/ET recombination technology, and recombinant rPRV SC-UL36-EGFP was rescued successfully. At last, the single viral particles with green fluorescent were monitored retrograde moving in the axon with an average velocity of 0.71 ± 0.43 μm/s at 0.5–2 h post infection (hpi) and anterograde moving with an average velocity of 0.75 ± 0.49 μm/s at eight hpi. Integration of fosmid library and Red/ET recombination technology in our work was highly efficient and stable for constructing PRV recombinants. This study will accelerate understanding the biology of PRV and the development of novel vaccines.

## Introduction

Pseudorabies virus (PRV) is a neurotropic herpesvirus, a member of *Alphaherpesvirinae* subfamily (Kramer et al., 2011). PRV invades the axon-endings distributed in the epithelial tissue after infection and replication at the epithelial cells. In detail, membrane fusion initiated between PRV envelope membrane and nerve cell membrane facilitates PRV to enter into neurons. Immediately, PRV virions undergo axonal retrograde transport from the axon to soma side and replicate in the nucleus. Subsequently, progeny virions are assembled and transported through the nervous system. Meanwhile, latency can also develop at the cell body in the neuron (Koyuncu et al., 2013). Due to the latent infection, it is very difficult to eradicate PRV. The direct contact between diseased animals and healthy animals is the main transmission route for this disease (Pomeranz et al., 2017). Clinical symptoms of PRV-infected pigs vary by age. Fattening pigs mainly suffer from respiratory disorders, while nursery pigs and younger piglets die from central nervous system (CNS) disorders (Pomeranz et al., 2005).

After PRV particle binding to axonal terminal, viral envelopes of PRV fuse with the plasma membrane of host cells. Then, viral particles enter into the axon where most tegument proteins are released into the cytoplasm. However, the inner tegument proteins such as UL37, UL36, UL21, UL16, UL14, US3, and ICP0 remain associated with capsids, and this capsid tegument complexes are transported toward nucleus where progeny viral genome is replicated (Luxton et al., 2005; Radtke et al., 2010). Several tegument proteins such as UL36, UL37, and UL21 play an essential role in recruiting motors for viral capsid tegument complexes for retrograde transport (Douglas et al., 2004; Luxton et al., 2006). UL36 and UL37 proteins interact with each other to form a physical complex (Roberts et al., 2009); on one hand, UL36 is the critical protein that directly binds to the dynein motor subunit p150 and p50, on the other hand, UL37 renders PRV neuroinvasion and retrograde axonal transport (Richards et al., 2017). Viral particles are unable to retrograde transport when UL36 is deleted. The deletion of UL37 does not show the same result as the UL36; UL37-deleted PRV is defective for secondary envelopment, which reduces capsid transport (Schipke et al., 2012). UL21 is another protein associated with neuroinvasion. It can promote PRV retrograde transport through interaction with cytoplasmic dynein light chain Roadblock-1 (Yan et al., 2019); however, UL21 mutation causes a delay in the spread to presynaptic neurons and reduces infectious particle production (Curanovic et al., 2009).

US9 is the key factor for recruiting KIF1A to promote anterograde transport which also needs gE and gI as helper proteins. Additionally, UL36 and US11, as candidates of PRV viral proteins, could recruit kinesin motors to envelop viral particles for anterograde transport. So UL36 interacts with motor proteins of opposite polarity and transport viral particles in both the anterograde and retrograde directions (Diefenbach et al., 2002). Fundamentally, the visualization of axonal transportation is the most convincing strategy to access this process, and the possible idealized marker locus is UL36 due to its direct interaction with molecular motor dynein (Zaichick et al., 2013).

For neurotropic herpesvirus, viral particles tagged with fluorescent protein have proven to be a powerful tool to visualize virus replication cycle and transport in long-distance of neurons (Kramer et al., 2012; Hogue et al., 2014). Since PRV is a linear, double-stranded DNA virus of about 143 kb, it is not easy to alter such a big genome. At present, several genetic manipulation methods have been used to modify the genome of PRV, including homologous recombination, bacterial artificial chromosome (BAC), and CRISPR/Cas9 (Lerma et al., 2016; Hubner et al., 2018). These methods were time-consuming and labor-intensive due to a series of plaque purification or were genetically unstable. Recently, the fosmid library for PRV TJ strain has been established by Zhou et al. (2018) and successfully constructed the rPRV TJ-VP26-EGFP. It was easily operated and highly efficient (Zhou et al., 2018). Therefore, the fosmid library is relatively dynamic at present due to the high recombination efficiency and the needlessness of purification (Zhou et al., 2018).

Extensive vaccination with Bartha-K61 vaccine strain has controlled PRV epidemics in China. Nevertheless, the disease still continue to circulate in China, especially after the emergence of variant PRV (An et al., 2013). All known full-length PRV sequences from the GenBank were divided into two clades according to ML phylogenetic tree analysis, and all the strains isolated from China were grouped in one clade (He et al., 2019). For the variant strain PRV TJ as an example, sequence comparison revealed that about 62 of total 67 viral proteins displayed variations compared with the previous isolates, and there are 43 viral proteins different compared with PRV ShuangCheng (SC) (Luo et al., 2014). To explore the characteristics of PRV, a convenient and efficient gene manipulation platform for PRVs is essential, especially for studying the evolution of PRV. Fosmid library is a convenient tool with numerous advantages.

In this study, a fosmid library for PRV SC was constructed. Using the fosmid library as well as Red/ET-mediated recombination, a recombinant rPRV SC-UL36-EGFP with EGFP fused into the amino-terminal of UL36 was successfully generated. It can help to visualize both anterograde and retrograde transport in neural circuits without affecting virus replication. This work will accelerate the progress of the PRV study and the development of novel vaccines.

## Materials and Methods

### Cells and Virus

The PRV SC strain (GenBank accession number: KT809429.1) is a virulent strain isolated in China in 1980, which is a reference challenge virus to evaluate the efficacy of the Bartha-K61 vaccine in China (Yuan et al., 1987). Porcine Kidney 15 cell (PK-15, ATCC CCL-33) and Vero cell lines were maintained in Dulbecco’s modified minimum essential medium (DMEM) (Gibco, Grand Island, NY, United States) containing 10% fetal bovine serum (FBS), 100 U/ml penicillin, and 100 μg/ml streptomycin.

### Extraction of Pseudorabies Virus Genomic DNA

The method of extraction of PRV full-length genomic DNA has been described previously (Smith and Enquist, 1999). PK-15 cells in 10 75-cm^2^ flask were infected with PRV SC strain at a multiplicity of infection (MOI) of 5 and cultured for 12–15 h at 37°C with 5% CO_2_. When all the cells show cytopathic effects (CPEs), the cells were collected, then washed with phosphate-buffered saline (PBS) twice. The cell pellet was lysed with 10 ml of LCM buffer [130 mM KCl, 30 mM Tris (pH 7.4), 5 mM MgCl_2_, 0.5 mM EDTA, 0.5% Non-idet P-40 (NP-40), and 0.043% 2-mercaptoethanol] to release viral capsids. Capsids were extracted with Freon twice and separated by ultracentrifugation through glycerol step gradients (8 ml of 5% glycerol and 16 ml of 45% glycerol) at 28,000 rpm for 2.5 h at 4°C. The genomic DNA was released by 10% sodium dodecyl sulfate (SDS) treatment. Then the DNA deposits were washed with 75% ethanol twice and dissolved in ultrapure water. The concentration of DNA was detected by the NanoDrop^TM^ 2000 (Thermo Scientific, Carlsbad, CA, United States) and gI gene was amplified using the primers listed in the Table 1 for sequencing. To check the infection activity, the genomic DNA was transfected into Vero cells using X-treme GENE HP DNA transfection reagent (Roche, Mannheim, Germany) according to manufacturer’s instructions.

**TABLE 1:** Primers for construction and identification of rPRV SC-UL36-EGFP.

Names	Sequences (5′–3′)	Target gene
	CGTGTCCAAATAAAAAGATTTTTCCCCCAC	
UL36-rpsl-F	GCGCGTGTGTTATTTCAGCCGGCCTGGTGA	
	TGATGGCGGGATCG TCGGGGTCATACTGATTACGATAGCCGACG	rpsLneo
UL36-rpsl-R	ACCACCGCGTCGGCCGTCATTCAGAAGAA CTCGTCAAGAAGGCGCGTGTCCAAATAAAA AGATTTTTCCCCCAC	
UL36-EGFP-F	GCGCGTGTGTTATTTCAGCCATGGTGAGCA	
	AGGGCGAGGAGCTG TCGGGGTCATACTGATTACGATAGCCGACG	EGFP
UL36-EGFP-R	ACCACCGCGTCGGCCGTCATTCTAGATCCG GTGGATCCCGGGCC	
JC-UL36-F	CGGCGAGCGTCAACGTGCGCGA	
JC-UL36-R	AGCTCGCTGATGGCGCACATG	rpsLneo/EGFP
gl-F	ATGATGGTGGCGCGCGACGTGA	gl
gl-R	TTATTGTTCTTCTGCGATGGTG	

### Construction of a Fosmid Library

Twenty micrograms of highly infective genomic DNA of PRV SC was pipetted using 200 μl tip to shear the DNA. The sheared genomic DNA was end-repaired using the End-Repairing Enzyme Mix to generate 5′ phosphorylated DNA. The DNA fragment was analyzed on a 1% gold agarose gel by pulsed-field gel electrophoresis (PFGE), a Lambda DNA Mono Cut Mix (New England BioLabs, Beijing, United Kingdom) as a size marker. DNA fragments ranging from 33 to 49 kb were excised from the gel, recovered using GELase (Epicentre, Madison, United States) and ligated into the pCC1FOS cloning-ready vector at room temperature for 4 h. The ligation mixture was packaged using MaxPlax Lambda Packaging Extracts and transformed into EPI300-T1^R^ cells. Except genomic DNA, all the materials and protocol as mentioned above were supplied by the Copy Control^TM^ Fosmid Library Production Kit (Epicentre, Madison, WI, United States). Two hundred clones were picked randomly and cultured overnight in 5 ml of LB liquid medium containing 12.5 μg/ml chloramphenicol and 50 μl of auto-induction solution (Epicentre). The fosmids were extracted using ZR BAC DNA Miniprep Kit (Zymo Research, United States), and the terminal of DNA fragment in the fosmids was sequenced based on pCC1FOS sequencing primers. The whole genomic DNA of PRV SC was assembled according to the terminal sequence of DNA fragment.

### Rescue of the Recombinant Pseudorabies Virus ShuangCheng

Thirteen fosmids that cover the whole genome of PRV SC in five groups (Tables 2, 3) were transfected into Vero cells cultured in 10-cm plates. Five fosmids (2 μg each) in each group were gently mixed together with 30 μl X-treme GENE HP DNA transfection reagent in 1 ml DMEM, incubated for 30 min at room temperature, and added into Vero cells. Before transfection, Vero cells were washed twice with PBS, 10 μg genomic DNA of PRV SC was transfected using the same way as a positive control, and non-transfected cells served as a negative control. CPEs in the Vero cells were observed at 5–7 days post-transfection, and cell supernatants were collected and stored for further identification of the recombinant viruses.

**TABLE 2:** Fosmids that cover the whole genome of PRV SC.

Fosmid	Location in genome(nt)	Size(bp)
60	1–33361	33361
142	1–31701	31701
22	1–31943	31943
50	29470–63289	33819
147	24017–61243	37226
78	29619–60993	31374
38	59987–92335	32348
66	50966–83734	32768
21	50301–99259	48958
24	80390–114445	34055
20	74192–109646	35454
65	111583–142825	31242
82	106487–142825	36338

**TABLE 3:** Fosmid groups that cover the genome of PRV SC.

Group	Combinations	CPE
1	60 + 50 + 38 + 24 + 65	+
2	142 + 147 + 66 + 20 + 82	+
3	22 + 78 + 66 + 24 + 65	+
4	60 + 147 + 21 + 24 + 65	+
5	22 + 50 + 38 + 24 + 65	+

### Electron Microscopy

The second generation (F2) of rPRV SC from the five fosmid groups and wild-type PRV SC were negatively stained with 2% phosphotungstic acid, and the morphologies of both Res-PRV SC and its parental virus PRV SC were observed under the electron microscope.

### Immunofluorescence Assay

After infection, the cells were fixed with ethanol at −20°C for 30 min, then washed with PBS three times. The fixed cells were incubated with swine anti-PRV sera (1:200 dilution) for 2 h at 37°C and then incubated with Alexa 488-conjugated goat anti-pig IgG (Thermo Fisher Scientific) (1:1,000 dilution) for 1 h at 37°C. After incubation, the cells were washed three times. The images were captured using an Olympus CK40 microscope.

### Restriction Fragment Length Polymorphism Analysis

PRV SC genomic DNA was purified as described by Smith and Enquist (1999). For RFLP analysis, the genomes of the viruses were digested with *Bam*HI (Thermo Fisher Scientific) in reaction mixtures containing 2 μg DNA, 10 × reaction buffer, 10 U restriction enzyme, and ddH_2_O in a total volume of 50 μl, and the reaction was performed at 37°C for 4 h. The digested samples were resolved in 0.8% agarose gel containing 0.2 μg/ml ethidium bromide and 1 × TAE buffer at 2 V/cm for 4 h at room temperature.

### One-Step Growth Curve and Plaque Size Determination

One-step growth curve was performed to compare the growth kinetics of the rPRV SC and parental virus PRV SC. PK-15 cells (or Vero cells) cultured in 24-well plate were infected with each virus at an MOI of five, then the supernatants and cells from three wells were harvested at 4, 8, 12, 24, 36, 48, 60, 72 h post infection (hpi) and stored at −80°C. The viral titers were determined by 50% tissue culture infectious dose (TCID_50_). To compare the plaque size of the rPRV SC and parental virus PRV SC, PK-15 cells (or Vero cells) were infected with each virus diluted serially in 10-fold and incubated for 1 h, then the medium was removed, and the cells were overlaid with 1% low-melting-point agarose diluted in DMEM. For each virus, 30 plaques were randomly selected, and their size was determined by ImageJ software.

### Red/ET-Mediated Recombination

The fosmid 50 (containing UL36 gene) was modified using the Red/ET-mediated recombination Counter Selection BAC Modification Kit (Gene Bridges, Berkeley, CA, United States) according to the manufacturer’s instructions. At first, the fosmid 50 and the Red/ET expression plasmid (pRed/ET) were co-transformed into competent *E. coli* DH10B cells by electroporation, naming with *E. coli* DH10B-fos50-Red/ET. Then the selection antibiotic cassette (rpsL-neo) flanked by 50 bp homology arms was transformed into competent *E. coli* DH10B-fos50-Red/ET to get *E. coli* DH10B-fos50-UL36-rpsL-Red/ET in which the rpsL-neo gene was fused to the amino-terminal of the UL36 gene. Finally, the EGFP flanked by 50-bp homology arms was transformed into competent *E. coli* DH10B-fos50-UL36-rpsL-Red/ET to get *E. coli* DH10B-fos50-UL36-EGFP in which the rpsL-neo gene fused to the amino-terminal of UL36 gene was replaced by EGFP. The modified UL36 ORFs were amplified and sequenced. The modified fosmid 50-UL36-EGFP was extracted from *E. coli* DH10B-fos50-UL36-EGFP and transfected into Vero cells with other fosmids to rescue the virus rPRV SC-UL36-EGFP. All the primers used in this experiment were listed in Table 1.

### PCR and Real-Time PCR

The genomic DNA was extracted from the cells and tissues infected with PRV as the template of PCR and real-time PCR amplification. The genomic DNA of PRV SC strain was used as the positive control and water as the negative control. The EGFP gene of rPRV SC-UL36-EGFP was amplified using forward primer EGFP-F and reverse primer EGFP-R, and gI genes of PRV SC, rPRV SC, and rPRV SC-UL36-EGFP were amplified by forward primer gI-F and reverse primer gI-R (listed in Table 1). The reaction mixture and condition were performed according to the manufacturer’s instruction of LA Taq^TM^ with GC Buffer (TaKaRa, Japan). Additionally, gI gene also was detected using forward primer gI-F and reverse primer gI-R and the Taq Man probe FAM-PRV-Cla (FAM-50-CGC GTG CAC CAC GAG GCC TT-30-BHQ1) in a real-time PCR as Meng et al. (2016) reported.

### Western Blot

PK-15 cell monolayers infected with rPRV-SC were gently washed with PBS and lysed by NP40 lysis buffer containing 1% PMSF (Solarbio, Beijing, China) for 1 h at 4°C. The lysates were mixed with 5× sample loading buffer, incubated in boiling water baths for 10 min, and cleared by centrifugation at 10,000 × *g* for 5 min at 4°C. A total of 30-μl sample was separated by SDS-polyacrylamide gel electrophoresis (PAGE) and transferred onto nitrocellulose membranes. After blocking with 5% skim milk for 2 h at 37°C, the membranes were incubated at room temperature for 2 h with specific mouse anti-gI polyclonal antibody (a gift from Jin Tian, State Key Laboratory of Veterinary Biotechnology, Harbin Veterinary Research Institute, Chinese Academy of Agricultural Sciences, Harbin, China), mouse anti-gB monoclonal antibody (a gift from Jing Zhao, State Key Laboratory of Veterinary Biotechnology, Harbin Veterinary Research Institute, Chinese Academy of Agricultural Sciences, Harbin, China), and anti-EGFP polyclonal antibody (Solarbio, Beijing, China). After washing membranes using PBST (PBS with 5% Tween 20) buffer, the membranes were incubated with DyLight 800 goat anti-mouse IgG (1:8,000; Thermo Fisher Scientific) at 37°C for 45 min, the membranes were washed three times in PBST, then visualized and analyzed with an Odyssey infrared imaging system (LI-COR Biosciences, Lincoln, NE, United States).

### Primary Neuronal Culture and Microfluidic Chambers

Dorsal root ganglia (DRG) from the spine of new-born BALB/c mice less than 3 days were collected, washed, and digested by Collagenase/Dispase (Roche) at 37°C in the CO_2_ incubator for 45–60 min. The neurons were separated from each other sufficiently, filtered, washed, and cultured in Neurobasal Medium (Gibco) supplemented with 100 ng/ml nerve growth factor 2.5S (Invitrogen), 2% B27 (Gibco), and 1% penicillin and streptomycin with 2 mM glutamine (Invitrogen). Before the neuron was planted in one side of the microfluidic chamber, the coverslips were treated with Poly-DL-ornithine hydrobromide (Sigma) for one night and laminin (invtrogen) for at least 6 h and washed with Hanks’ balanced salt solution (HBSS) buffer two times, dried completely, then covered with microfluidic device, neurons were added in one well of microfluidic device, flowed into the chamber connected with two adjacent wells, after 3 days culturing, the axons grow to another chamber along the chamber microgrooves (see illustration). Infection was performed by replacing the Neurobasal Medium in the distal wells and changed with 10^7^ TCID_50_ rPRV SC-UL36-EGFP. Time-lapse imaging was achieved by automated sequential capture.

### Pseudorabies Virus Infection of Mice

Thirty-five 6-week-old specific pathogen-free (SPF) BALB/c mice were randomly divided into seven groups, five mice in each group. Groups 1–3 were each intramuscularly injected with 100 μl of different titer (10^2^–10^4^ TCID_50_) of PRV SC strain at left hind limb, and groups 4–6 were inoculated with rPRV SC-UL36-EGFP strain at the same way, viral titer, and position; group seven as control was injected with 100 μl DMEM. The clinical signs were recorded every day until 7 days post infection (dpi), and the 50% lethal doses (LD_50_) of PRV SC and rPRV SC-UL36-EGFP strains in mice were calculated. The tissue samples from brain, spinal cord, sciatic nerve, leg, and foot of mice infected with 10^4^ TCID_50_ of PRV SC or rPRV SC-UL36-EGFP were collected to detect the level of PRV genome using real-time PCR following the method of Meng et al. (2016).

### Ethics Statement

The Animal Ethics Committee approval number is Heilongjiang-SYXK-2006-032. All the experiments were operated in the Biosafety Level II laboratory following strict biosecurity measures according to instructions of the Harbin Veterinary Research Institute. Fertilized BALB/c mice for DRG collection were bred.

### Statistical Analysis

All experiments were performed at least three times. Data were statistically analyzed by unpaired t-tests and a one-way ANOVA in Prism 7.0 software (GraphPad Software, La Jolla, CA, United States). Differences were considered significant if there was an unadjusted *P*-value less than 0.05. The instantaneous velocity and direction were analyzed by Imaris × 64 9.3.1 software (Imaris Software, Oxford Instruments, United Kingdom), and the data were analyzed by Statistical Product and Service Solutions (SPSS) 13.0 software (SPSS Software, IBM, United States).

## Results

### Construction of the Fosmid Library for Pseudorabies Virus ShuangCheng

**FIGURE 1:**
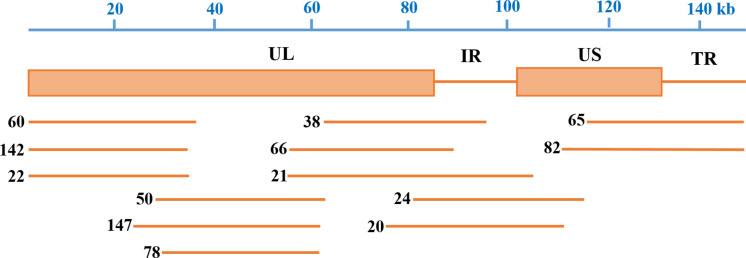
Fosmid library construction for the pseudorabies virus ShuangCheng (PRV SC) strain. Genomic structure of PRV and the five fosmid DNAs used for the rescue of PRV. Blue numbers show the location of each fosmid fragment in the PRV genome, and the new names are the ID of each fosmid.

After end-blunting and phosphorylation, the DNA fragments were cloned into the fosmid vectors and transformed into *E. coli*. A total of 200 clones were randomly picked from the fosmid library for end-sequencing. As a result, there are 165 clones match for PRV SC genome according to the two terminal sequences of each DNA fragment, and a majority of which contains insert of 30–49 kb. Moreover, the fosmid library can cover the complete genome of PRV SC. There are three DNA fragments including 5′ terminal sequence, two DNA fragments including 3′ terminal sequence, most DNA fragments including the middle part of genome, and some fragments including incomplete terminal genome. At last, 13 fosmids were selected for generating the fosmid combinations that cover the complete genome of PRV SC (Table 2). Five sets of overlapping fosmid combinations were prepared to rescue the recombinant PRV SC (rPRV SC); each group consisting of five overlapping fosmids (Figure 1 and Table 3).

### Rescue and Characterization of Recombinant Pseudorabies Virus ShuangCheng Based on Overlapping Fosmids

**FIGURE 2:**
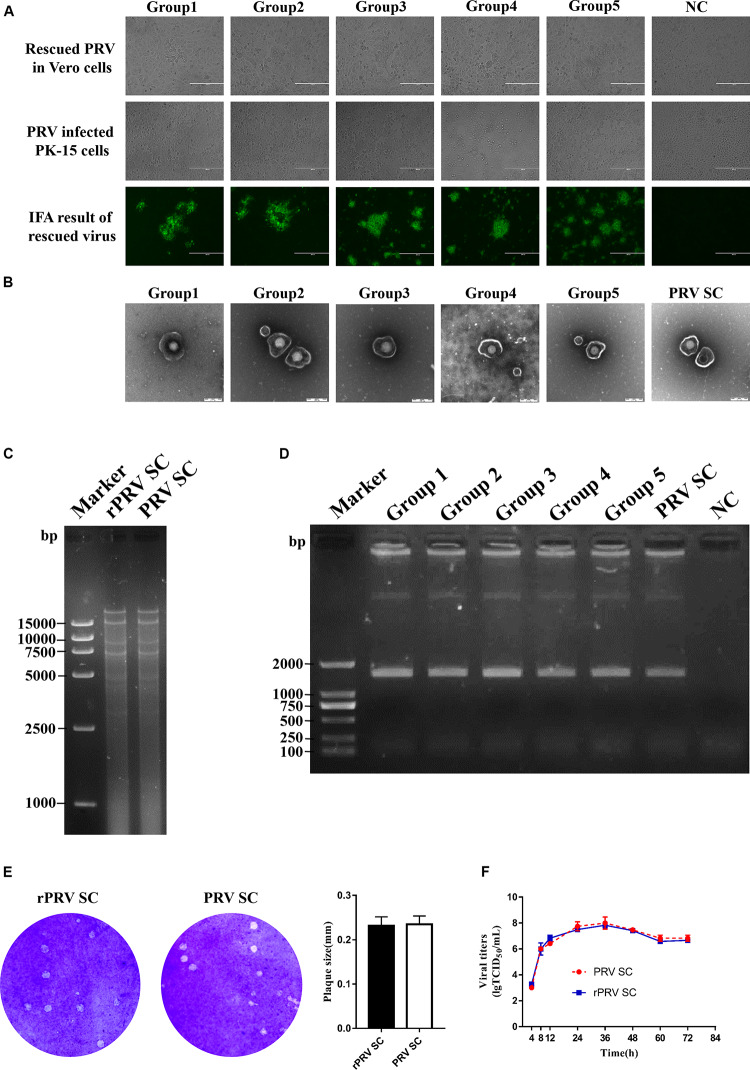
Identification of recombinant pseudorabies virus ShuangCheng (rPRV SC). **(A)** The first row represents the cytopathic effects (CPEs) of Vero cells, which is infected with the first generation of rPRV SC, and no infection as negative control (NC). The second row represents the CPEs of PK-15 cells infected with the first generation of rPRV SC from different groups in the second line. The third row represents the immunofluorescence assay (IFA) of PK-15 cells infected with rPRV SC using an anti-PRV serum. The images were captured using EVOS Cell Imaging Systems under 10 × magnification. **(B)** Transmission electron of PRV SC and rPRV SC. The virus particles were 25,000 times magnified. **(C)** The restriction fragment length polymorphism analysis of the rescued and the parent PRV in 0.8% agarose. The genome of the rescued and the parent PRV was digested with *Bam*HI. **(D)** PCR amplification of the gI gene from the genome of PRV SC and rPRV SC. **(E)** Plaque of PRV SC and rPRV SC in the PK-15 cells. The diameter of plaques was averaged from three independent experiments. **(F)** One-step growth curve of PRV SC and rPRV SC in the PK-15 cells.

Five sets of fosmid plasmids were transfected into Vero cells to rescue rPRV SC; non-transfected Vero cells served as the negative control. CPEs of five transfection groups were observed at 5–7 dpi, while no CPEs in the negative control during the progress. This suggested that all the CPEs were caused by the rPRV SC, not by contaminated PRV or other viruses. Then Vero cells and PK-15 cells were infected with the first generation of rPRV SC. After culturing for 48 h, CPE and immunofluorescence assay (IFA) were performed, and the results were consistent with the first-generation virus (Figure 2A). To check the morphology of recombinant viral particles, under the electron microscope, the rPRV SC particles showed similar to that of the parental virus with an apparently external envelope (Figure 2B). To further identify the genome of rPRV SC, we performed PCR using the specific primer for gI gene. As a result, the gI genes of PRV SC was successfully amplified from the genomic DNA of every rPRV SC and wild PRV SC (Figure 2D). To further confirm rPRV SC, the genome of rPRV SC from group five was digested with *Bam*HI, the result of restriction fragment length polymorphism (RFLP) showed no significant difference from parental PRV SC (Figure 2C). Moreover, the comparison of the replication kinetics and plaque morphology of rPRV SC with parental PRV SC showed no significant difference in the PK-15 cells (Figures 2E,F). So, the results demonstrated that rPRV SC could be rescued successfully using the five sets of fosmid plasmids.

### Generation and Identification of Recombinant Pseudorabies Virus ShuangCheng-UL36-Enhanced Green Fluorescent Protein

**FIGURE 3:**
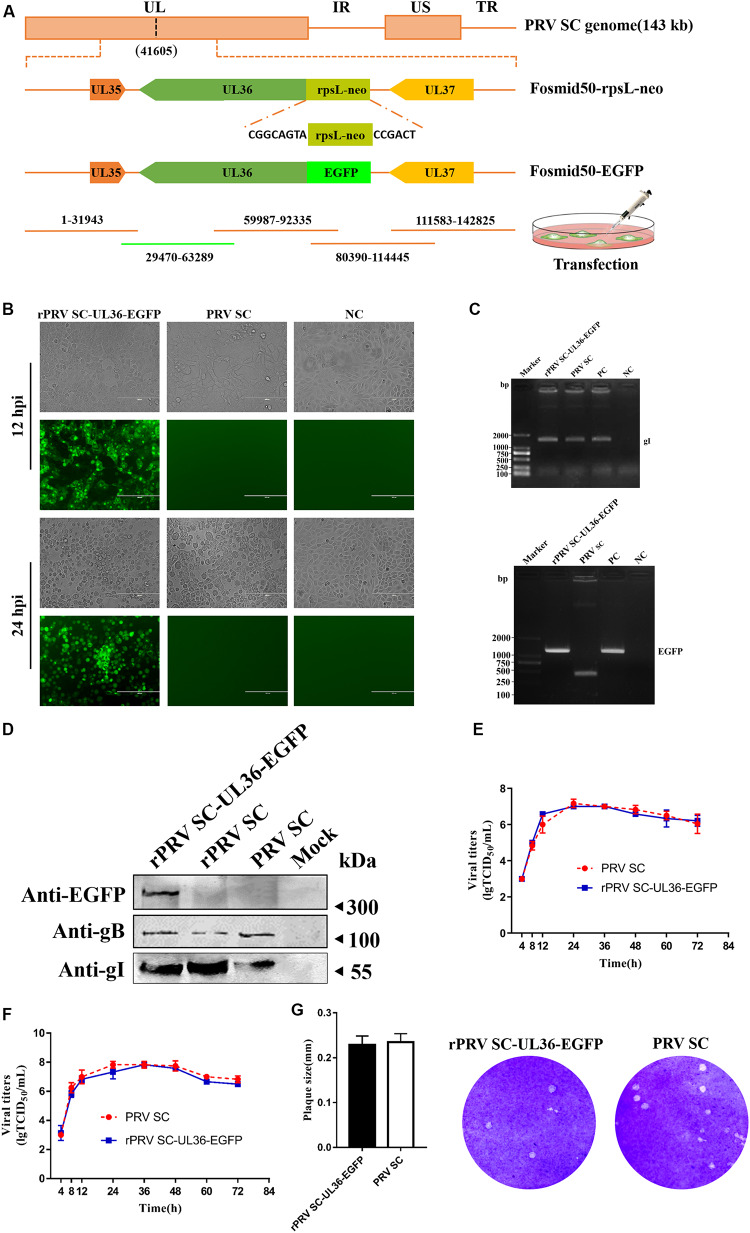
Rescue and identification of recombinant pseudorabies virus ShuangCheng (rPRV SC)-UL36-enhanced green fluorescent protein (EGFP). **(A)** Schematic for rPRV SC-UL36-EGFP design. Finally, Fosmid 22(1-31943), 38(59987-92335), 24(80390-114445), 65(111583-142825), and Fosmid 50-EGFP were transfected into Vero cells to rescue rPRV SC-UL36-EGFP. **(B)** The green fluorescence and cytopathic effects (CPEs) in the PK-15 cells infected with rPRV SC-UL36-EGFP or PRV SC at 12, 24 h post infection (hpi). **(C)** The viral gene gI and EGFP from rPRV SC-UL36-EGFP and PRV SC were identified by PCR. Plasmid pflag-gI and pok-UL36-EGFP act as a positive control. **(D)** The expression of gB, gI, and EGFP in the rPRV SC-UL36-EGFP- and PRV SC-infected cells were identified by Western blot at 12 hpi. **(E)** One-step growth curves of rPRV SC-UL36-EGFP and PRV SC in Vero cells. **(F)** One-step growth curves of rPRV SC-UL36-EGFP and PRV SC in PK-15 cells. **(G)** Plaque size of rPRV SC-UL36-EGFP and PRV SC. The diameter of 30 pots was measured.

UL36, a large tegument protein, attaches to the capsid during axonal retrograde transport. Previous studies have shown that UL36 directly interacts with dynein subunit p150 and p50 to mediate PRV retrograde transport (Zaichick et al., 2013). Consequently, UL36 is a possible ideal reporter target for visualization axonal retrograde kinetics. To rescue rPRV SC-UL36-EGFP, EGFP was fused into the amino-terminal of UL36. Fosmid 50 (29470-63289) that harbors UL36 gene was modified by the Red/ET-mediated recombination (Figure 3A). The modified fosmid 50 (fosmid 50-UL36-EGFP) and other fosmid plasmids in group five were transfected into Vero cells. After 5 days’ incubation, CPEs with green fluorescence were observed, and the supernatant was inoculated into PK-15 cells. The images of CPEs with green fluorescence were captured at 12 and 24 hpi in rPRV SC-UL36-EGFP-infected cells, and only CPEs were observed in the PRV SC-infected cells (Figure 3B). The gI and EGFP gene were amplified from the genomic DNA of the rPRV SC-UL36-EGFP and PRV SC strains, and the EGFP gene could be amplified only from the rPRV SC-UL36-EGFP genome but not from PRV SC (Figure 3C). To further identify rPRV SC-UL36-EGFP, the expression of gB, gI, and EGFP proteins were identified by Western blotting. The result showed the same bands of gB and gI presented in the samples of rPRV SC-UL36- EGFP-, rPRV SC-, and PRV SC-infected groups, and UL36-EGFP was detected only in rPRV SC-UL36-EGFP-infected group (Figure 3D). In addition, rPRV SC-UL36-EGFP and PRV SC showed similar growth kinetics in both PK-15 and Vero cells (Figures 3E,F), and the plaque size of rPRV SC-UL36-EGFP was also the same as that of PRV SC (Figure 3G).

### Pathogenicity of Recombinant Pseudorabies Virus ShuangCheng-UL36-Enhanced Green Fluorescent Protein Strain in Mice

To check the pathogenicity of rPRV SC-UL36-EGFP strain compared with the wild strain PRV SC, mice were infected with same titer of rPRV SC-UL36-EGFP as PRV SC simultaneously. All the mice in the control group remained healthy during the experiments. The mice infected with 10^4^ TCID_50_ of both rPRV SC-UL36-EGFP and PRV SC strain showed serious itching at 2 dpi; the skin around of injected position was scratched and bitten intervally at the beginning, more and more frequently, until the mice could not move and died as Brittle et al. (2004) described. The itching symptoms of mice infected with 10^3^ TCID_50_ started at 3 dpi. The depression and rough hair could be observed in most of the mice infected with 10^2^ TCID_50_. When the mice suffered from itching, they would die at 10–12 h later. So the average time point of itching onset was earlier about half a day than the average mortality time point as shown in Table 4. The LD_50_ of rPRV SC-UL36-EGFP (1.48 × 10^3^ TCID_50_) was lower than that of wild PRV SC strain (2.09 × 10^3^ TCID_50_), but there was no statistically significant difference. So the pathogenicity of rPRV SC-UL36-EGFP was the same as that of its parental virus PRV SC.

**TABLE 4:** Result of mouse infected with PRV SC and rPRVSC-UL36-EGFP strain.

Groups	Amounts	Dose (TCID_50_)	Clinical signs	Morbidity (mean days of itch onset)	Mortality (mean days to death)
PRVSC	5	10^4^	+++	5/5 (2.6)	5/5 (3.1)
	5	10^3^	++	3/5 (3.25)	3/5 (3.75)
	5	10^2^	+	0/5	0/5
rPRVSC-UL36-EGFP	5	10^4^	+++	5/5 (3.1)	5/5 (3.7)
	5	10^3^	++	3/5 (3.3)	3/5 (3.9)
	5	10^2^	+	1 (4.5)	1/5 (5)
DMEM	5	100 μL	/	0/5	0/5

*Mean time to itch onset and death was calculated for five mice of each group after intramuscular injection with different dose of viruses shown in the table. Clinical symptoms were scored in four degrees:+++, severe; ++, moderate; +, mild; /, none.*

**FIGURE 4:**
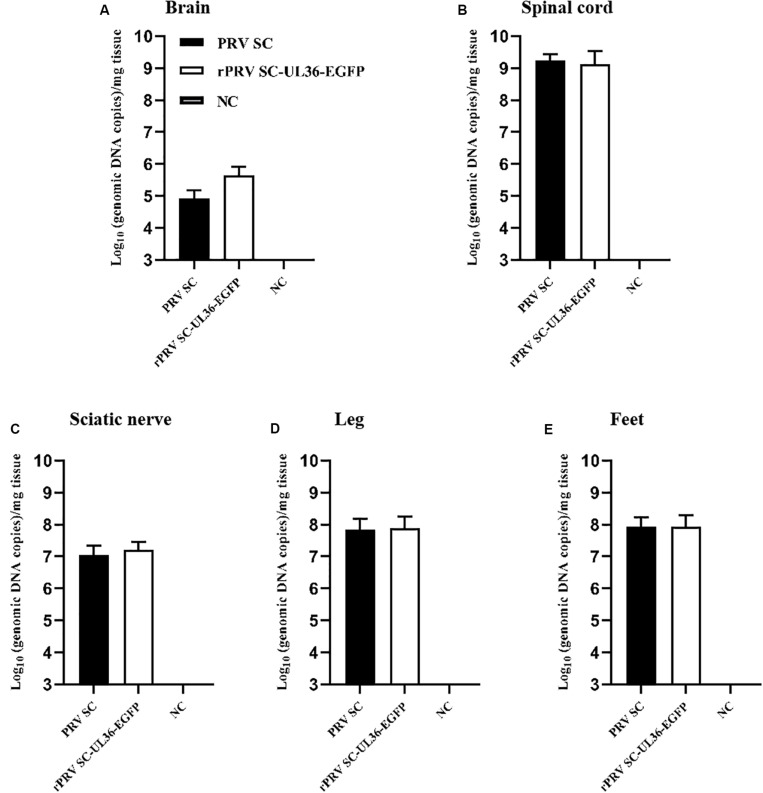
Viral loads in the tissue of mice infected with pseudorabies virus ShuangCheng (PRV SC) and recombinant PRV SC (rPRV SC)-UL36-enhanced green fluorescent protein (EGFP) strains. The indicated tissues of mice that were mock infected or infected with 10^4^ TCID_50_ PRV SC or rPRV SC-UL36-EGFP strains, the gI gene was detected by real-time PCR to quantify genomic DNA of both two PRV strains. The data represent average copies + SE (error bars) for five samples per group. Samples of 100 mg brain **(A)**, 100 mg lumbar spine **(B)**, and the sciatic nerves **(C)**, 100 mg leg muscles **(D)**, and feet **(E)** on the inoculated side were harvested from each mouse after they died of infections.

The genomes of both PRV SC and rPRV SC-UL36-EGFP strains were detected by real-time PCR to compare the viral replication in the injection point and nervous system. As a result, the viral genomic DNA was positive in the sciatic nerve (1.6 × 10^7^ and 1.6 × 10^7^ copies) (Figure 4C), spinal cord (1.7 × 10^9^ and 1.3 × 10^9^ copies) (Figure 4B), and brain (8.5 × 10^5^ and 4.4 × 10^5^ copies) (Figure 4A) where particles of PRV were retrograde transported and replicated. Moreover, the highest viral copies were in the lumbar spine, the peripheral nerve center near the injection point. Although all the mice infected with 10^4^ TCID_50_ of PRV SC and rPRV SC-UL36-EGFP strain died before 4 dpi, the virus titers were lower in the brain compared to the spinal cord and sciatic nerves. There were also high copies of viral genomic DNA in the legs (7.0 × 10^7^ and 7.6 × 10^7^ copies) (Figure 4D) and the feet (8.4 × 10^7^ and 8.7 × 10^7^ copies) (Figure 4E) where PRV particles were anterograde transported to the nerve endings. The viral loads in the tissue of mice infected with rPRV SC-UL36-EGFP strain were not statistically different from that of the PRV SC strain. In conclusion, the EGFP fused with UL36 gene of PRV SC strain did not affect viral replication and spread in the nervous system of mouse.

### Visualization of the Enhanced Green Fluorescent Protein-Tagged Viral Particle Transport in Neuron

**FIGURE 5:**
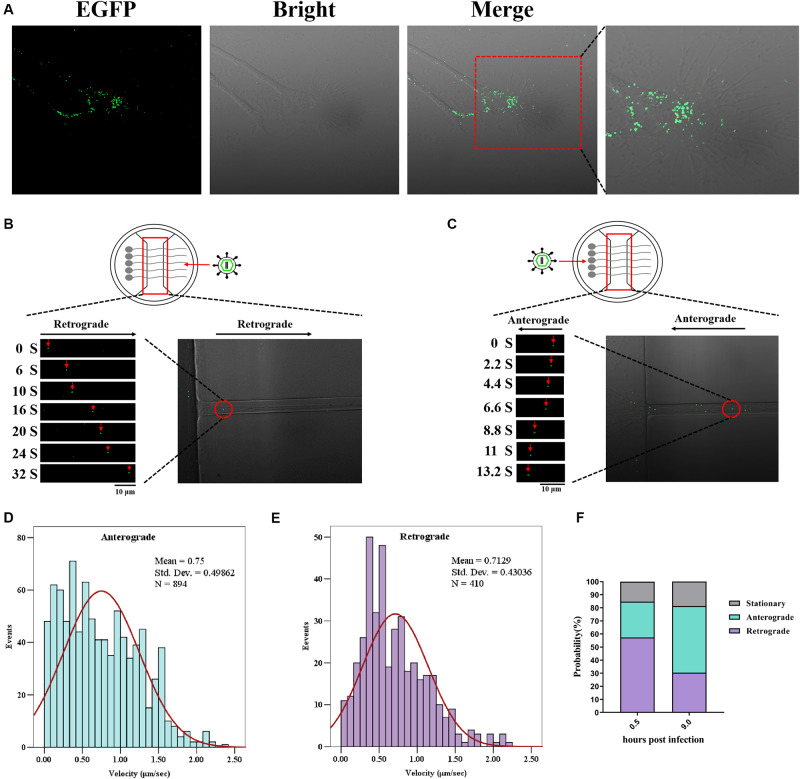
Enhanced green fluorescent protein (EGFP)-tagged capsid moving in the neuron. Dorsal root ganglia (DRG) were plated in the main channel of Microfluidics. After 3 days, neurons were infected with recombinant pseudorabies virus ShuangCheng (rPRV SC)-UL36-EGFP at a multiplicity of infection (MOI) of 10 at the axon side. Visualization of rPRV SC-UL36-EGFP action by Time-lapse was performed at 0.5 h post infection (hpi). **(A)** A large number of viruses attaching to the axon endings were captured. **(B)** The retrograde of single rPRV SC-UL36-EGFP particle along the axon was tracked from 0 to 32 s. **(C)** The anterograde of single rPRV SC-UL36-EGFP particle along the axon was tracked from 0 to 13.2 s. **(D)** Instantaneous velocity distributions of retrogradely moving capsids. **(E)** Instantaneous velocity distributions of anterogradely moving capsids. **(F)** Proportions of capsids in the three states including anterograde movement, retrograde movement, and stationary state.

Next, we checked whether the EGFP-tagged reporter virus rPRV SC-UL36-EGFP could be used to track the trajectory of PRV particle transport in the neuron and analyzed the velocity kinetics, direction, and displacement of transport. The rPRV SC-UL36-EGFP was added into two wells of the microfluidics and flowed into the chamber in the axonal side. In the first 10–20 min, the green particles randomly distributed in the medium; only a minority of viral particles that attached to the axon terminals were captured in the same focal length with the axons. At this time, viral particles were quiet at the vision of microscope as shown in Figure 5A. A little of the particles had entered into the axon, and maybe part of viral particles just attached to the upper axon not the end.

After membrane fusion, capsid–tegument complexes were started moving along the axon. There is more than one mode of movement after penetration of viral particles into the axon. Some viral particles in the terminal axon were moving toward the cell body (retrograde movement), here, the movement of one EGFP-tagged capsid was observed during 0–32 s (Figure 5B), but some capsids stopped halfway to the cell center (stationary). Other viral particles just moved back at one stage (anterograde movement) and forward at the next stage. The percentages of capsids in the three states including anterograde movement, retrograde movement, and stationary state were calculated as shown in Figure 5F. In detail, 57.3% of total capsids moved through the retrograde at 0.5 hpi, 27.4% of capsids moved through anterograde, and 15.3% remained in stationary state. So, majority of viral capsids moved toward the cell body after entering into the axon. The instantaneous velocity of retrograde movement varied from 0 to 2.22 μm/s, and the average velocity was 0.71 ± 0.43 μm/s (Figure 5E).

The green fluorescence particles retrograde transport intermittently only happened at the first 0.5 to 2 h. The time duration varied based on the length and number of axons. Another continual particle movement can be observed until 8 hpi; the virus run away from the cell body (anterograde) reverse to the first 2 h observed, the movement of one particle from 0–13.2 s was shown in Figure 5C. The capsids also perform three states at this time, including anterograde movement, retrograde movement, and stationary state. The percentages of each state were 50.9%, 30.4%, and 18.7%. So half of them moved far away from the cell body. The instantaneous velocity of anterograde movement varied from 0 to 2.35 μm/s, and the average velocity was 0.75 ± 0.49 μm/s (Figure 5D).

Overview of the speed distribution of anterograde and retrograde shows that the forward velocity is widely distributed between 0 and 1.5 μm/s, while the reverse velocity tends to the normal distribution curve (Figures 5D,E). Subsequently, the distribution of virus particles’ state in the axon shows that the viral particles mainly conduct retrograde transport at 0.5–2 hpi and mainly conduct anterograde transport at about eight hpi (Figure 5F). Those results indicate that rPRV SC-UL36-EGFP was suitable for tracing PRV behavior between cell body and axon in the neuron.

## Discussion

The technology for constructing recombination PRV develops quickly in recent years. CRISPR/Cas9 is applied, which leads to the gene deletion mutation easier than before, but the method cannot be used in the gene insertion mutation, especially specific site mutation and seamless insert (Xu et al., 2015; Tang et al., 2016). Meanwhile, plaque purification of recombination PRVs from parental viruses always takes more than 1 month. Infectious BAC of PRV, another genetic manipulation technology, is efficient for gene insertion and deletion using Red/ET technology in *E. coli* (Osterrieder et al., 2003; Wang et al., 2018). However, it takes several months to construct BAC clones, but the genetic and phenotypic change occurs in the recombinant virus due to the unstable large genome of PRV in *E. coli*. Fosmid library is a powerful platform for rescuing DNA viruses and has been used in genomic manipulation of DNA viruses to generate a duck enteritis virus vaccine strain (Chen et al., 2013). The first fosmid library for pseudorabies variant strain PRV TJ has been reported in 2018 by our group (Zhou et al., 2018). In this study, a fosmid library for pseudorabies strain PRV SC was established using the same method. In this study, we got 200 fosmid DNAs that cover the whole genome of PRV SC. Most of the DNA segments are between 30 and 49 kb, and each fragment was cloned into pCC1FOS vector hosted in the *E. coli* strain separately. The positive percentage of most of the fragments was more than 90%. We selected 13 plasmids separated into five groups, five plasmids in one group, and all the groups can rescue the virus successfully. Further, we rescued the rPRV SC-UL36-EGFP strains. There is no significant difference between rPRV SC and PRV SC in growth kinetics, restriction profiles, plaque size, and morphology of virus particles, so the fosmid groups are highly efficient for recovering recombination PRV SC.

The capsid protein VP26 was fused usually with fluorescent protein such as EGFP, Red, or mCherry as a tool for tracing PRV in neural circuits, but the amino-terminal of VP26 fused with EGFP would reduce virus replication by nearly 10-fold (Douglas et al., 2004; Krautwald et al., 2008). The recombinant virus with mCherry fusion to VP26 carboxy-terminal expresses more VP26 fusion protein in infected cells and incorporates more VP26 fusion protein into virus particles. Individual virus particle exhibits brighter red fluorescence compared to the amino-terminal mCherry–VP26 fusion virus (Hogue et al., 2018). However, the fluorescent protein fusion to VP26 of herpes simplex virus (HSV) is also reported to cause a reduction of the neurovirulence in mice (Krautwald et al., 2008).

UL36 is the critical protein that links PRV capsid with the dynein of the neuron. UL36 and UL37 form viral capsid–tegument complexes for retrograde transport *via* an amino-terminal domain. The carboxy-terminal of UL36 comprises a proline/alanine-rich region that plays an essential role in PRV replication (Bottcher et al., 2006). In this work, EGFP protein was fused to the amino-terminal of UL36 protein, and rPRV SC-UL36-EGFP performs the same morphology, growth kinetics, and plaque sizes with the parental virus; all these show EGFP protein fused at the amino-terminal of UL36 protein did not affect viral replication and also did not affect the pathogenicity to mouse. The green fluorescent-labeled capsid moving along the axon of neuron both anterograde and retrograde was monitored step by step, and the average velocities of retrograde and anterograde movements were 0.71 ± 0.43 and 0.75 ± 0.49 μm/s. This is close to 0.62 ± 0.18 μm/s of HSV anterograde movement in cortical neuron axons by the same statistical analysis method (Dong et al., 2020). However, The average of PRV movement speed was 1.2 μm/s in the research by Hogue et al. (2018), while the events less than 0.5 μm/s were deleted in the data of Gaussian distribution. The velocity changed at different times post-infection (Dong et al., 2020). Moreover, the virus strain, the kind of neuron, and statistical analysis method may all affect the reported average speed of capsid transport in axon retrograde. So EGFP fusion to the amino-terminal of UL36 did not affect capsid–tegument complexes transport along the axon of neuron *in vitro*. Our result demonstrated that the rPRV SC-UL36-EGFP could be used for tracking PRV movement in the neuron.

In summary, this fosmid library was efficient and flexible for rescuing recombination PRV SC. The fosmid library and Red/ET technology would be applied in other PRV strains and other DNA virus research. We successfully rescue the rPRV SC-UL36-EGFP using this method. Moreover, we found that the EGFP fusion to the amino-terminal of UL36 did not affect viral replication and could also work as a tool for tracing PRV in neural circuits.

## Data Availability Statement

All datasets generated for this study are included in the article/supplementary material.

## Ethics Statement

The animal study was reviewed and approved by The Animal Ethics Committee approval number is Heilongjiang-SYXK- 2006-032.

## Author Contributions

YS and H-JQ designed the research. HQ and MA performed the experiments. HW wrote the manuscript. All authors reviewed the manuscript.

## Conflict of Interest

The authors declare that the research was conducted in the absence of any commercial or financial relationships that could be construed as a potential conflict of interest.
